# Supplementary material for: Evaluating Alternative Correction Methods for Multiple Comparison in Functional Neuroimaging Research
Source: Brain Sci. 2019 Aug 12;9(8):198. doi: 10.3390/brainsci9080198 (PMC6721788; doi:10.3390/brainsci9080198)
Supplement: Supplementary file 1 [file brainsci-09-00198-s001.pdf]

Supplementary Materials

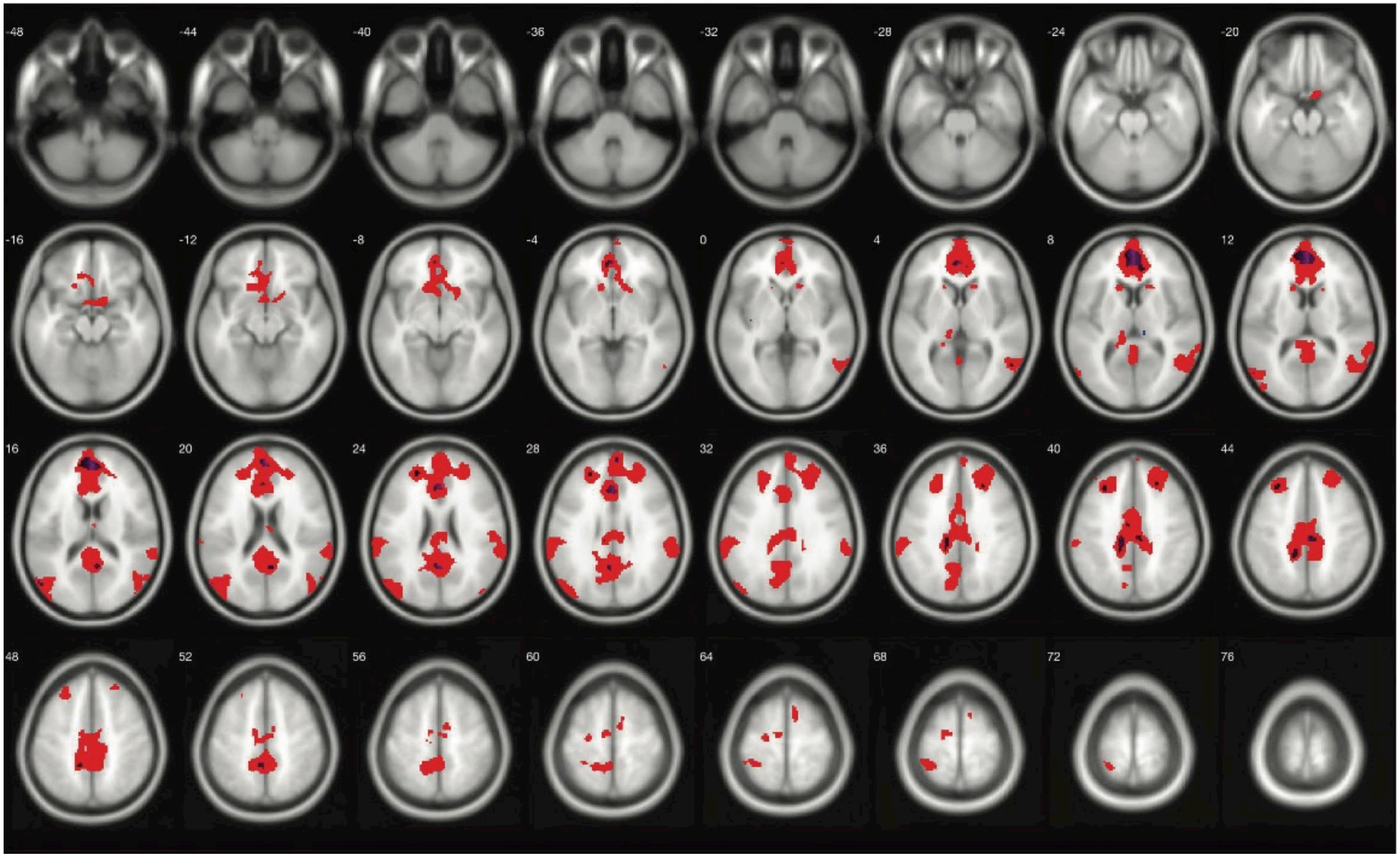

Figure S1. Voxels survived SnPM thresholding with moral psychology fMRI data (Red to purple: SnPM clusterwise, Blue to purple: SnPM voxelwise)

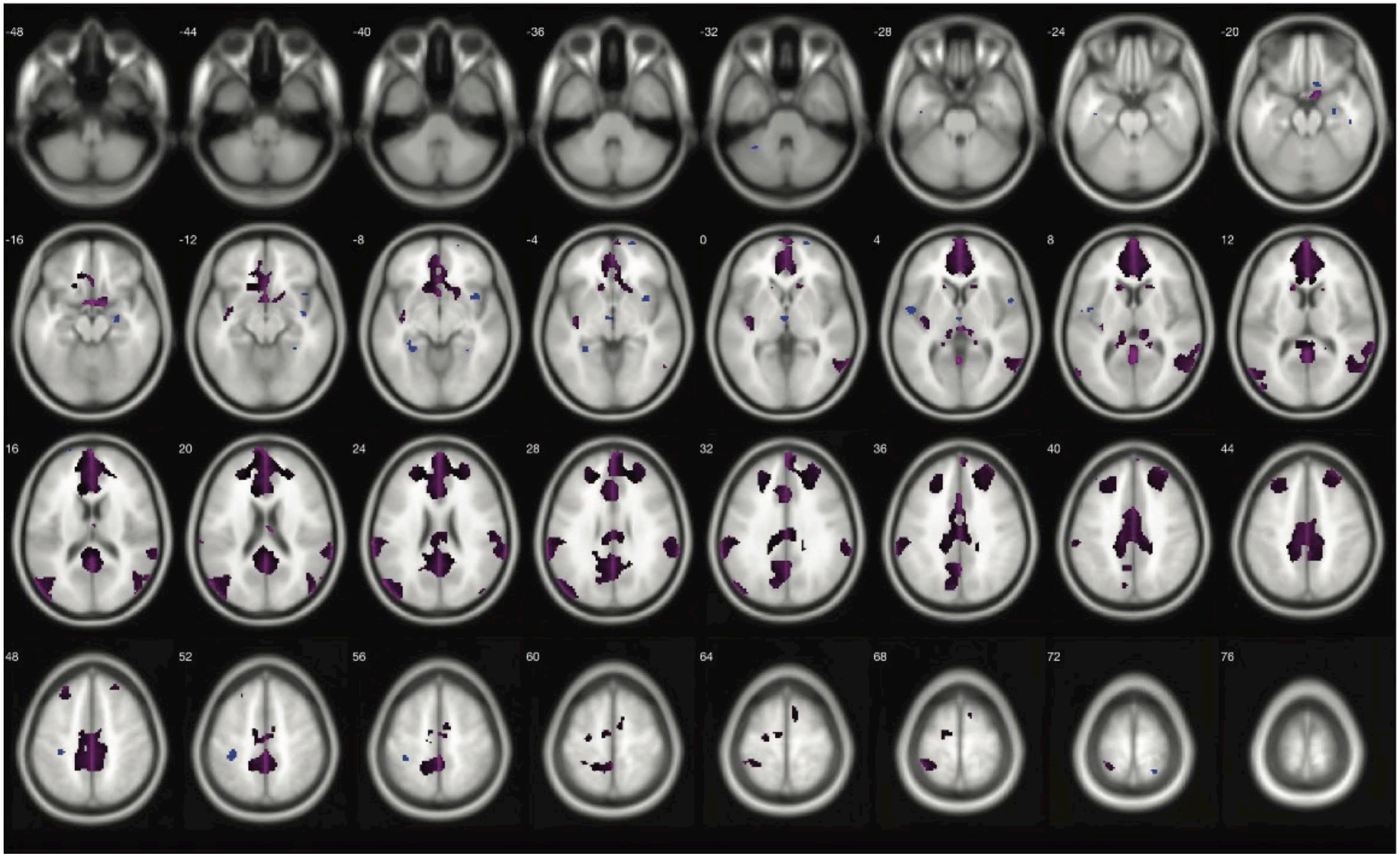

Figure S2. Voxels survived 3DClustSim thresholding with moral psychology fMRI data (Red to purple: with acf, Blue to purple: without acf)

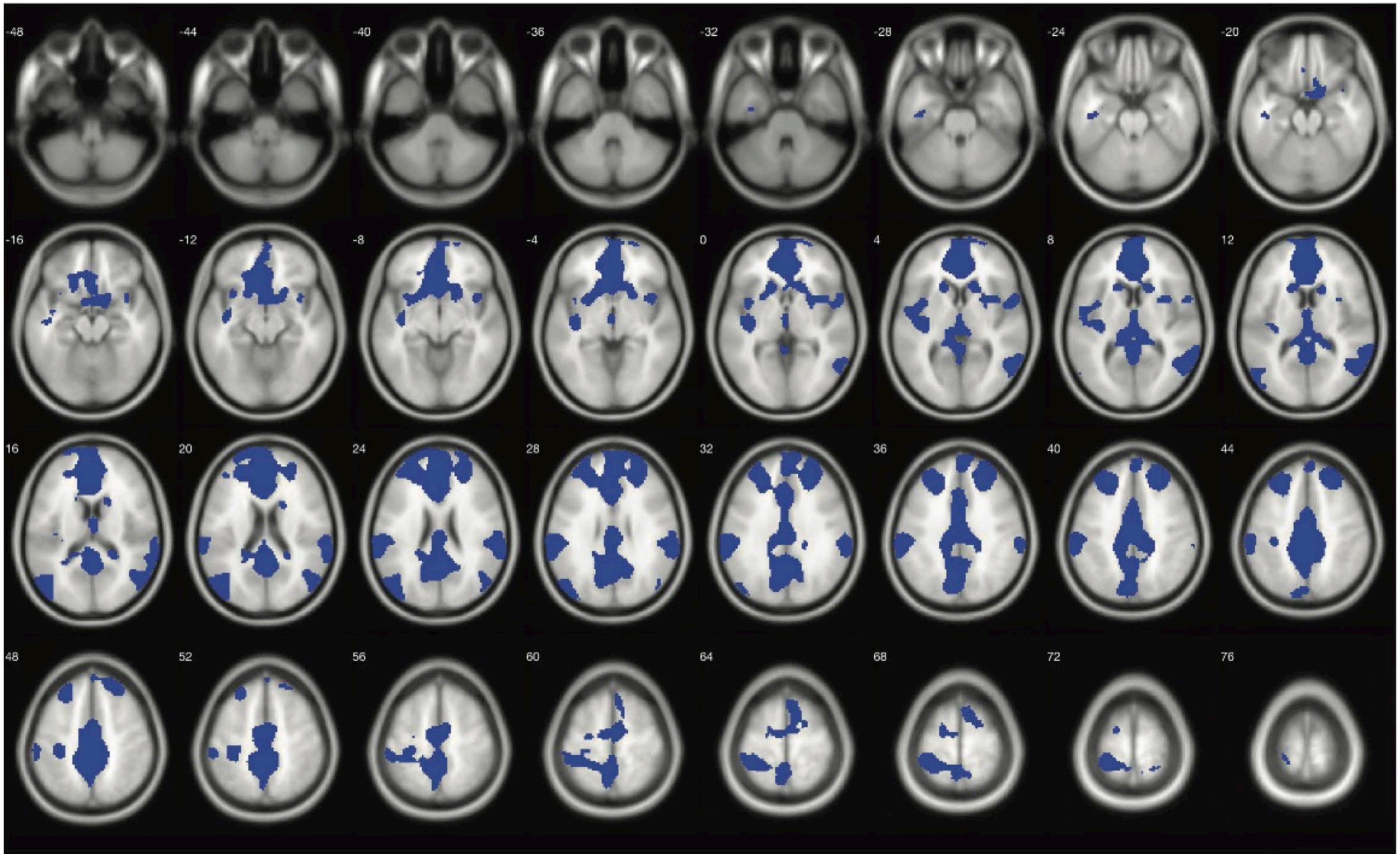

Figure S3. Voxels survived TFCE thresholding with moral psychology fMRI data

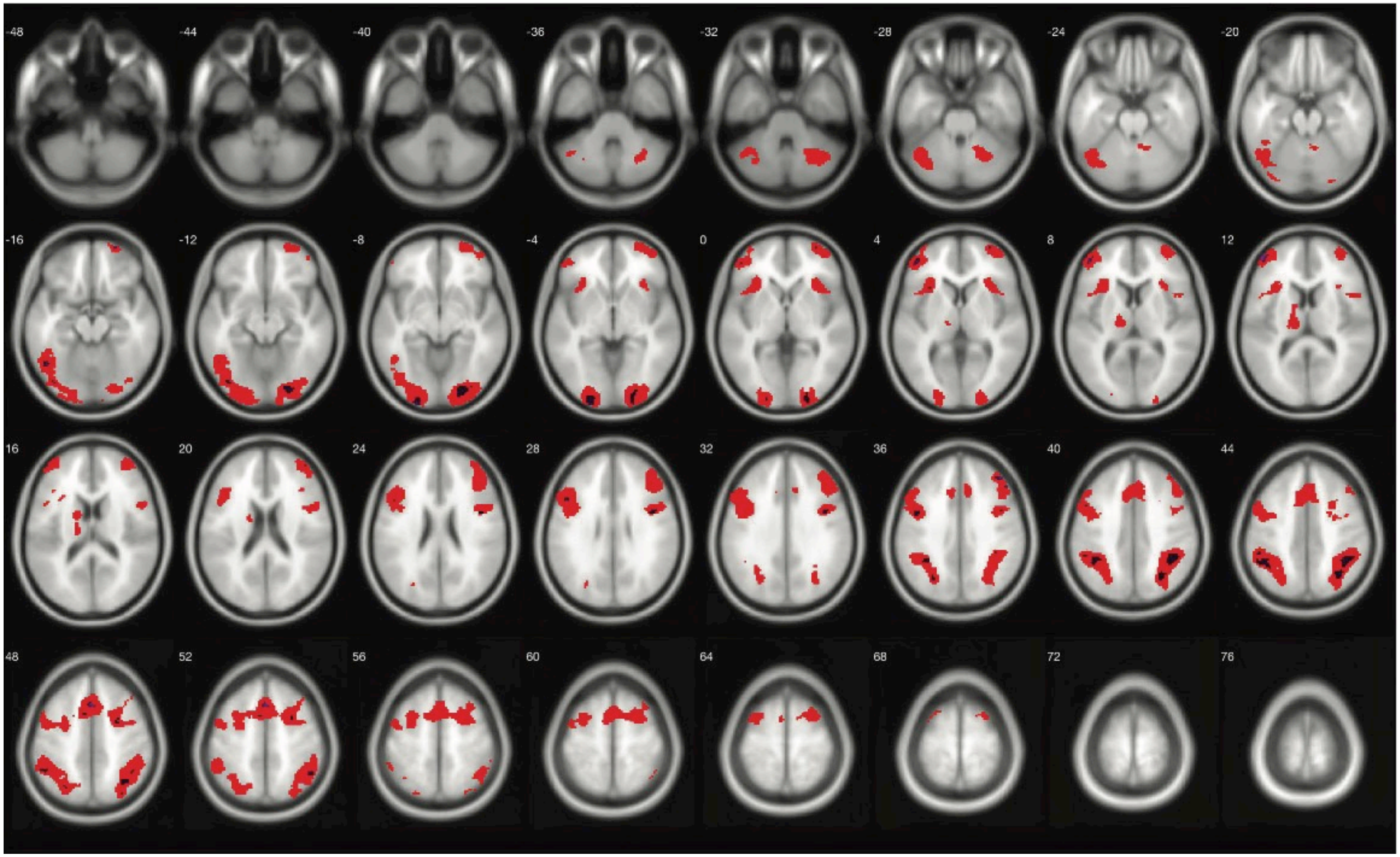

Figure S4. Voxels survived SnPM thresholding with working memory fMRI data (Red to purple: SnPM clusterwise, Blue to purple: SnPM voxelwise)

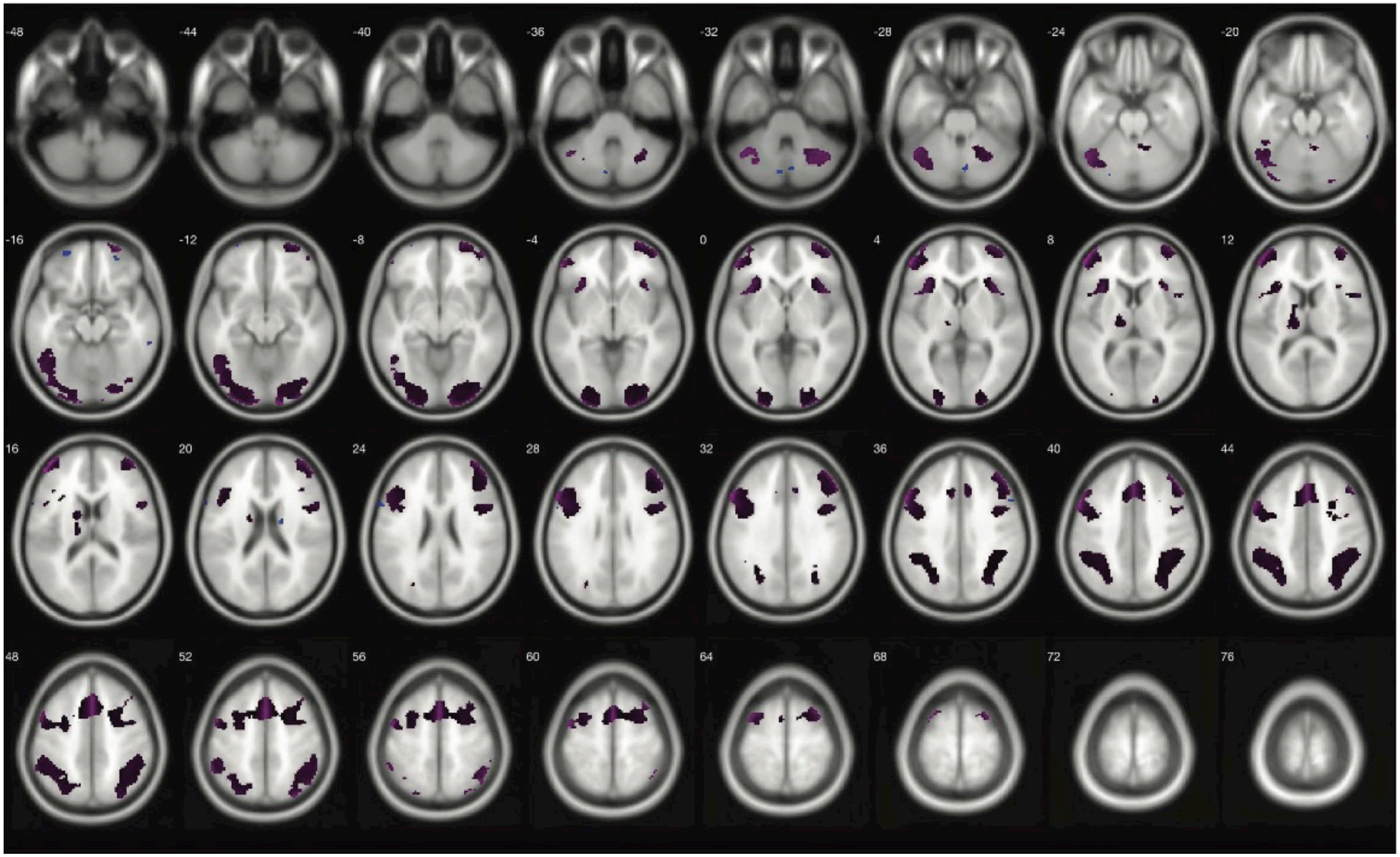

Figure S5. Voxels survived 3DClustSim thresholding with working memory fMRI data (Red to purple: with acf, Blue to purple: without acf)

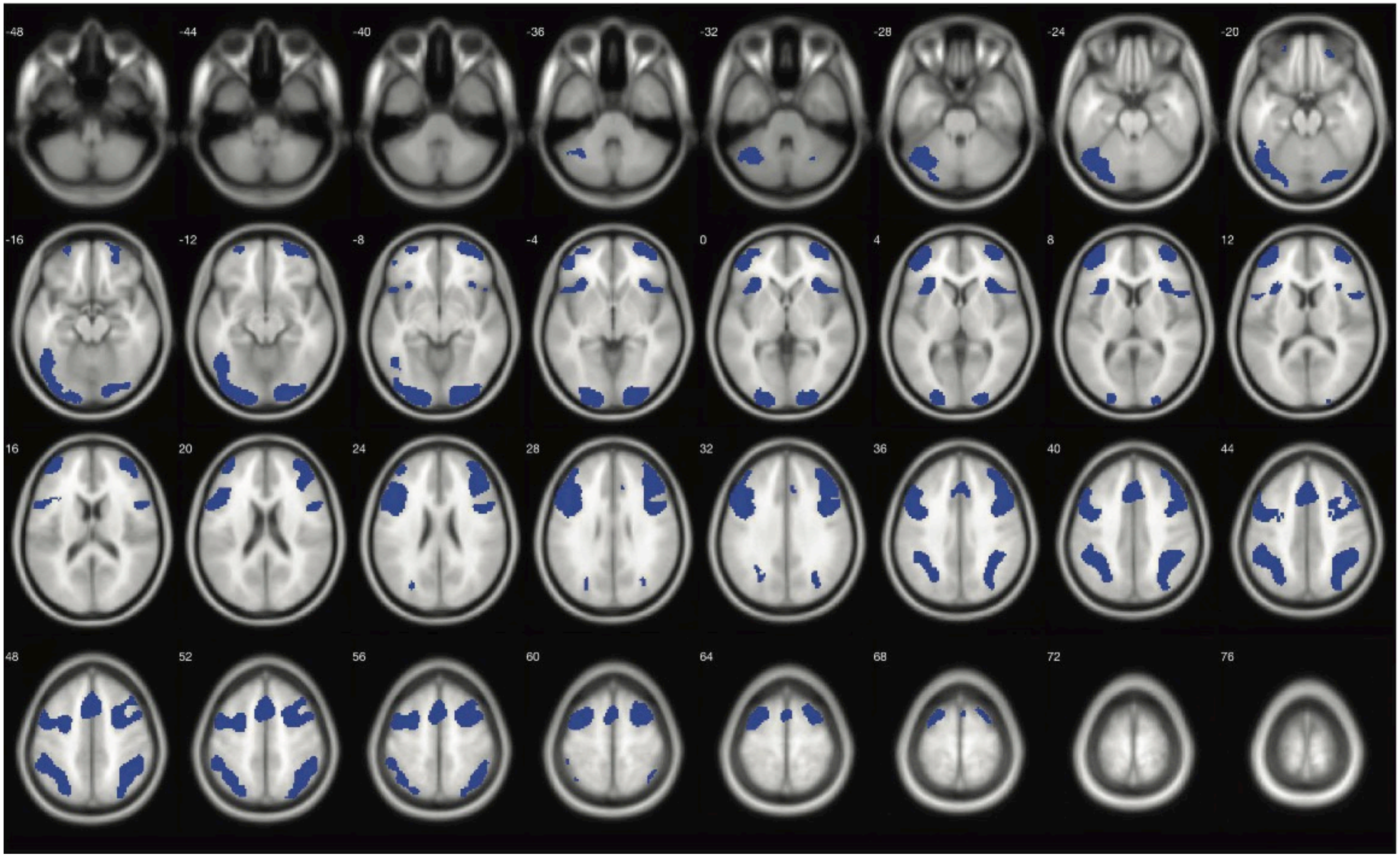

Figure S6. Voxels survived TFCE thresholding with working memory fMRI data
